# MLL-AF4 upregulates 5-lipoxygenase expression in t(4;11) leukemia cells via the ALOX5 core promoter

**DOI:** 10.3389/fphar.2024.1520507

**Published:** 2025-01-14

**Authors:** Marius Hyprath, Maximilian Molitor, Ilona Schweighöfer, Rolf Marschalek, Dieter Steinhilber

**Affiliations:** ^1^ Institute of Pharmaceutical Chemistry, Goethe University, Frankfurt, Germany; ^2^ Institute of Pharmaceutical Biology, Goethe University, Frankfurt, Germany

**Keywords:** 5-lipoxygenase, MLL, MLL-AF4, leukemia, leukocyte, leukotriene

## Abstract

5-Lipoxygenase (5-LO), encoded by the gene *ALOX5*, is implicated in several pathologies. As key enzyme in leukotriene biosynthesis, 5-LO plays a central role in inflammatory diseases, but the 5-LO pathway has also been linked to development of certain hematological and solid tumor malignancies. Of note, previous studies have shown that the leukemogenic fusion protein MLL-AF4 strongly increases *ALOX5* gene promoter activity. Here, we investigate the upregulation of *ALOX5* gene expression by MLL-AF4. Using reporter assays, we first identified the tandem GC box within the *ALOX5* promotor sequence as the main target of MLL-AF4. Subsequently, we narrowed down the domains within the MLL-AF4 protein responsible for *ALOX5* promoter activation. Our findings indicate that MLL-AF4 binds to the *ALOX5* promoter via its CXXC domain and that the AF9ID, pSER and CHD domains redundantly activate transcriptional elongation. Knockdown of the MLL-AF4 gene in the human B cell line SEM revealed that MLL-AF4 is an inducer of *ALOX5* gene expression in leukemic cells with lymphoid properties. Finally, we found that the MLL-AF4-related protein MLL-AF9, a driver of acute myeloid leukemia, similarly acts on the *ALOX5* promoter. Taken together, we show that two prominent MLL fusion proteins are *ALOX5* gene inducers in cells with lymphoid features.

## Introduction

The 5-lipoxygenase (5-LO) enzyme fulfills several cellular functions. First, it is well known as the pivotal enzyme in the biosynthesis of leukotrienes (Rådmark et al., 2015). Moreover, recent studies have shown that the protein elicits further non-canonical cellular functions as regulator of gene expression which interferes with β-catenin/Wnt and TGFβ signaling (Rådmark et al., 2007; Brand et al., 2018; Kreiß et al., 2022). Moreover, 5-LO can interact with the RNA-processing enzyme dicer, and thus, interferes with microRNA maturation and processing (Provost et al., 1999; Uebbing et al., 2021). Pathophysiologically, the 5-LO pathway is implicated in inflammatory reactions, but it is also known that high 5-LO expression correlates with the development of solid tumors as well as leukemogenesis (Moore and Pidgeon, 2017; Göbel et al., 2023; Claesson et al., 2024). Obviously, canonical and non-canonical 5-LO functions provide advantages for tumors regarding growth and progression (Kahnt et al., 2024).

The *ALOX5* gene is located on chromosome 10 and spans a genomic range of around 82 kilobases (kb). The *ALOX5* promoter structure has been analyzed in several studies, and binding sites for several proteins in transcriptional regulation have been found within a core region ∼800 bp from the translation start site (TSS) (Funk et al., 1989; In et al., 1997). In summary, *ALOX5* gene expression is regulated in a complex manner via regulatory sequences controlling the initiation of transcription and others in distal gene regions regulating transcription elongation (Stoffers et al., 2010). Reporter gene studies revealed that the fusion protein MLL-AF4, a product of the leukemogenic chromosomal rearrangement of the genes KMT2A (MLL1) and AFF1 (AF4), induces *ALOX5* core promoter activity by more than 40-fold (Ahmad et al., 2014; Ahmad et al., 2015). The MLL1 (mixed lineage leukemia, MLL) protein is a histone lysine N-methyltransferase and is encoded by the KMT2A gene (histone-lysine N-methyltransferase 2A) on chromosome 11q23. It serves as a platform for protein complexes involved in reading and writing of chromatin epigenetic modifications that regulate gene transcription. AF4 is encoded by the AFF1 gene (ALF transcription elongation factor 1) on chromosome 4 and serves again as a platform to form the multi-protein super elongation complex (SEC) (Benedikt et al., 2011; Marschalek, 2016). The rearrangements of chromosomes 4 and 11 results in two mutant chromosomes known as derivative chromosome 4 (der4) and derivative chromosome 11 (der11), encoding the fusion proteins MLL-AF4 and AF4-MLL, respectively. This rearrangement is one of the most prominent events in the onset of acute lymphoblastic leukemia (ALL) which is found in 5%–10% of all leukemia patients (Behm et al., 1996; Winters and Bernt, 2017). In addition, it is diagnosed as sole genetic aberration in 80% of all infant ALL cases (Meyer et al., 2023).

Given the prominent role of MLL-AF4 in leukemogenesis and its known activating potential on the *ALOX5* promoter, the present study elucidates the mechanism of this interplay.

## Materials and methods

### Cell lines and culture conditions

If not stated otherwise, all cell culture materials have been purchased from Thermo Fisher Scientific™ (Thermo Fisher Scientific™ Waltham, Massachusetts, United States). The adherent cell lines: HeLa (ACC 57, DSMZ, Hannover, Germany), HT-29 (ACC 299, DSMZ) and U-2 OS (HTB-96, ATCC, Manassas, United States) were cultured in a humidified atmosphere with 5% CO_2_ at 37°C in Dulbecco’s modified Eagle´s medium without phenol red (wDMEM). The medium was supplemented with 10% fetal bovine serum (FBS, Capricorn Scientific GmbH, Ebsdorfegrund, Germany), 1 mM sodium pyruvate, GlutaMAX™, 100 U/mL penicillin and 100 μg/mL streptomycin. Cells were grown to 70%–90% confluency before being passaged (twice a week). The suspension cell lines MV4-11 (ACC 102, DSMZ) and SEM (ACC 546, DSMZ) were cultured in a humidified atmosphere with 5% CO_2_, at 37°C in RPMI 1,640 medium supplemented with 10% FBS, 100 U/mL penicillin and 100 μg/mL streptomycin. Cultures were split twice a week. Both cell lines were seeded at a concentration of 0.3 × 10^6^ cells/mL and 1.0 × 10^6^ cells/mL for routine culture, respectively.

### Plasmid design and cloning

A list of all DNA primer sequences and restriction enzymes used for cloning is provided in the supplementary materials. Restriction enzymes were purchased from New England Biolabs (New England Biolabs GmbH, Frankfurt am Main, Germany), DNA primers were received from Eurofins (Ebersberg, Germany). Promotor constructs were cloned using the NEBuilder HiFi DNA Assembly kit (New England Biolabs GmbH, Frankfurt am Main, Germany) and were introduced into DH5α *E. coli*. Vectors pGL3B and pRL-SV40 were purchased from Promega (Promega GmbH, Walldorf, Germany). The reporter construct containing 800 bp of the *ALOX5* core promoter (pGL3-ALOX5-0.8) and a corresponding deletion construct lacking a characteristic five-fold tandem GC-Box (pGL3-ALOX5-0.8-∆GC) were designed by our group and previously referred to as pN10 and pN10∆GC0 (Klan et al., 2003). MLL-AF4 expression vectors are based on the empty vector pTarget (Ahmad et al., 2014), which is referred to in the present study as VC (vector control). The MLL-AF4 domain constructs contained the following amino acid positions (AA) of the wildtype protein sequence: MLL-AF4_∆CHD AA 1–1,869 (∆AA 1,870–226); MLL_ALFpSER AA 1–1,537 (∆AA 1,538–2,226); MLL_ALF AA 1–1455 (∆AA 1,456–2,226); MLL_CHD AA 1–1,362, 1,871–2,226 (∆AA 1,363–1,870); N-MLL AA 1–1,362 (∆AA 1,363–2,226); MLL-AF4_CXXCmut AA 1,188 C→D; MLL-AF4_∆AT AA 1–169, AA 309–2,226 (∆AA 170–308); MLL-AF4_∆Men∆AT AA 1-1, AA 309–2,226 (∆AA 2–308); Men-CXXC-CHD AA 1–18, AA 1,148–1,203, AA 1,871–2,226 (∆AA 19–1147, ∆AA 1,204–1,870). The expression vector for MLL-AF9 (pT-MLL-AF9) was cloned using the described plasmid N-MLL. The C-terminal part of the AF9 sequence, containing the last 193 amino acids of the protein, were amplified from cDNA generated from the cell line MonoMac 6 that carries a translocation t (9;11) (p22;q23) (Super et al., 1995). Plasmids pSBtetGH and pSB100X were obtained from Eric Kowarz (Goethe University, Frankfurt, Germany) and were used for the generation of stably transfected cell lines overexpressing an inserted transgene after incubation with doxycycline (Kowarz et al., 2015). The coding sequence for MLL-AF4 was inserted into the pSBtetGH construct to generate the pSBtetGH_MLL-AF4 plasmid. The C-terminal tagged GFP constructs (Men-CXXC-CHD-GFP, N-MLL-GFP, MLL_CHD-GFP, MLL-AF4_CXXCmut-GFP or MLL-AF4-GFP) were cloned by using the mentioned untagged constructs and the coding sequence for EGFP. The sequence was obtained by using the Lonza (Basel, Switzerland) pMAX-GFP control vector.

### Generation of cell lines with inducible expression of MLL-AF4

Cell lines carrying a stably integrated, doxycycline-inducible expression system encoding MLL-AF4 were generated using the Sleeping Beauty transposon system (Kowarz et al., 2015). Plasmids employed were pSBtet-GH_MLL-AF4, encoding MLL-AF4, GFP and a hygromycin resistance marker, as described under plasmid design and cloning and SB100X encoding transposase (Kowarz et al., 2015). For transfection, HT-29 cells and U-2 OS cells (1 × 10^6^ and 0.3 × 10^6^ per well, respectively) were seeded into 6-well plates in 5 mL wDMEM. A total of 1900 ng pSBtet-GH_MLL-AF4, 100 ng SB100X and Lipofectamine™ LTX with Plus Reagent (Thermo Fisher Scientific™) were added to each well (4:1 ratio of Lipofectamine:DNA according to manufacturer’s protocol). After 24 h, the medium was replaced by selection medium, consisting of wDMEM supplemented with 500 μg/mL hygromycin B (Thermo Fisher Scientific™). Transfected cells were selected with hygromycin B under standard culture conditions (see cell lines and cell culture) for 3 weeks. Cells were sub cultured twice a week at 80% confluence. The cellular GFP signal was used to monitor the selection progress via fluorescence microscopy.

### Transient reporter gene assays

HeLa cells were seeded in 24-well plates at (0.5 mL wDMEM; density of 4 × 10^4^ cells/well) 24 h before transfection. Polyethyleneimine (PEI, Sigma-Aldrich, St. Louis, United States) was used as transfection agent. The DNA-PEI mix was prepared in medium free from serum and antibiotics (DNA:PEI ratio of 4:1). Each transfection mix contained 400 ng reporter plasmid (either pGL3B, pGL3-ALOX5-0.8, pGL3B-ALOX5-0.8-∆5GC, pGL3-TK or pGL3-TK-5GC), 200 ng expression plasmid or the corresponding empty vector (either VC, pT-MLL-AF4, pT-MLL_CHD, pT-N-MLL, pT-MLL-AF4_CXXCmut, pT-MLL-AF4_∆AT, pT-MLL-AF4_∆Men∆AT or pT-Men-CXXC-CHD) and 20 ng Renilla luciferase control plasmid (pRL-SV40). The transfection mix was incubated for 20 min at room temperature (RT) before adding 50 µL to the cells. After 16 h of incubation in a humidified atmosphere with 5% CO_2_, at 37°C, medium was replaced by fresh wDMEM. After further 24 h of incubation, the medium was removed and the cells were washed once with PBS. Luciferase luminescence was measured using the Dual-Glo^®^ Luciferase assay system (Promega Corporation, Fitchburg, United States) in Lumitrac™ 96 well plates (Greiner AG, Kremsmünster, Österreich) with a TECAN Spark^®^ plate reader (Tecan Group, Männedorf, Switzerland). Relative luminescence units (RLU) were calculated by normalizing Firefly luciferase LU to Renilla luciferase LU.

### Reporter gene assays with stably transfected cells

Stably transfected HT-29 or U-2 OS cells expressing MLL-AF4 (U-2 OS_MLL-AF4; HT-29_MLL-AF4) or the corresponding wildtype cells (U-2 OS_wt; HT-29_wt) were seeded in 24-well plates (0.5 mL wDMEM, 1.2 × 10^4^ cells/well for U-2 OS and 1.2 × 10^5^ cells/well for HT-29). After 24 h, cells were transfected with 600 ng reporter plasmid (either pGL3B, pGL3-ALOX5-0.8 or pGL3B-ALOX5-0.8-∆5GC) and 20 ng Renilla luciferase control plasmid (pRL-SV40) using Lipofectamine^®^ LTX&PLUS™ Reagent (Thermo Fisher Scientific™) at a LTX to Plus reagent ratio of 4:1. After 16 h, the medium was removed and replaced with wDMEM containing 1 μg/mL doxycycline. wDMEM without doxycycline served as a control. The cells were incubated for another 24 h, the medium was removed, and the cells were washed once with PBS. Luciferase activities were measured as described above for transient reporter assays.

### Analysis of subcellular localization

HeLa cells were seeded in 24-well plates (0.5 mL wDMEM; 1.5 × 10^4^ cells). After 24 h, cells were transfected with 620 ng of one of the following expressions constructs which encode full length MLL-AF4 or deletion mutants thereof (see “Cell lines and cell culture”), each fused with a C-terminal GFP tag (Men-CXXC-CHD-GFP, N-MLL-GFP, MLL_CHD-GFP, MLL-AF4_CXXCmut-GFP or MLL-AF4-GFP). The mentioned pMAX-GFP plasmid expressing GFP was used as a control. PEI reagent was used for transfection with a DNA:PEI ratio of 1:4. After 16 h, the medium was replaced with maintenance medium and cells were incubated for additional 24 h. Subsequently, cells were washed with PBS and were fixated with 4% paraformaldehyde (PFA, Sigma Aldrich) in PBS for 20 min at RT. PFA was removed, cells were washed with PBS and stained with 1 μg/mL 4′,6-diamidino-2-phenylindole (DAPI, Sigma Aldrich) in PBS for 20 min at RT. After a final washing step, cells were stored in PBS at 4°C until image acquisition. Pictures were captured with a Zeiss AX10 microscope attached to a Zeiss Axiocam 305 color imaging system (Carl Zeiss AG, Jena, Germany). An image overlay was generated using the ImageJ software (Schneider et al., 2012).

### cDNA synthesis and RT-qPCR

MV4-11 and SEM cells (0.2 × 10^6^ each) were harvested and RNA was isolated with the NucleoSpin RNA/Protein Mini Kit (Macherey-Nagel GmbH and Co. KG, Düren, Germany) following the manufacturer´s protocol. The RNA amount was determined by measuring the absorbance at 260 nm with a NanoDrop 2000 spectrophotometer (Thermo Fisher Scientific™). cDNA synthesis was performed using the HighCapacity RNA to cDNA kit (Thermo Fisher Scientific™) from 400 ng of RNA. qPCR was performed with 10 ng cDNA equivalents per well in MicroAMP^®^ FastAMP 96-well reaction plates (Thermo Fisher Scientific™) with PowerUP SYBR Green Master Mix (Thermo Fisher Scientific™). mRNA expression levels of the following target genes were analyzed by qPCR on a StepOnePlus™ Real-Time PCR-System (Thermo Fisher Scientific™) using the corresponding primer pairs (from Eurofins, Ebersberg, Germany): *ALOX5* (fwd: CTC​AAG​CAA​CAC​CGA​CGT​AAA, rev: CCT​TGT​GGC​ATT​TGG​CAT​CG), *UBC* (fwd: CTG​GAA​GAT​GGT​CGT​ACC​CTG rev: GGT​CTT​GCC​AGT​GAG​TGT​CT), *GAPDH* (fwd: GCA​TCC​TGG​GCT​ACA​CTG​A, rev: CCA​CCA​CCC​TGT​TGC​TGT​A), *MLL-AF4* (fwd: GGT​CCA​GAG​CAG​AGC​AAA​CAG, rev: TGT​ATT​GCT​GTC​AAA​GGA​GGC​G), *MLL-AF9* (fwd: TGG​TTT​GCT​TTC​TCT​GTC​GC, rev: GGA​CCT​TGT​TGC​CTG​GTC​TG. GAPDH served as housekeeping control, which was used to normalize the measured CT values and data are shown as relative induction compared to negative control (2^(-∆∆CT)^).

### Western blot analysis

For the analysis of cellular 5-LO protein expression, cells were seeded in 10 cm petri dishes in 10 mL DMEM supplemented with 1 μg/mL doxycycline at a density of 5 × 10^6^ cells per dish for HT-29_wt, and HT-29_MLL-AF4 and 2.5 × 10^6^ cells for U-2 OS_wt and U-2 OS_MLL-AF4. Parallel cultures without doxycycline served as a control. After 48 h of incubation, cells were harvested, suspended in SDS lysis buffer (77 mM SDS, 1.5 M Glycerol, 56 mM Tris, pH 6.8) and sonicated with an ultrasonic homogenizer at 10% of maximum amplitude (Sonopuls HD 200 with Sonopuls microtip MS72, BANDELIN electronic GmBH and Co. KG, Berlin, Germany). Cell lysates were centrifuged (10 min, 12,000 rcf, 4°C) and the supernatant was transferred to a fresh tube. Protein concentration was determined using the Pierce™ BCA Protein Assay Kit (Thermo Fisher Scientific™) and a Tecan Infinite M200 plate reader (Tecan Group Ltd.). 30 μg of total cellular protein per sample were separated by SDS-PAGE (10% running gel, 80 V for 15 min and 130 V for 100 min). Purified recombinant 5-LO protein served as a positive control and Precision Plus Protein™ All Blue Prestained Protein Standard (Bio-Rad, Hercules, United States) was used for size estimation. Separated proteins were transferred to 0.2 µm nitrocellulose membranes (Bio-Rad) with a wet tank method using a Mini Trans-Blot^®^ cell (Bio-Rad) (125 mA for 85 min). Membranes were blocked for 1 h using EveryBlot Blocking Buffer (Bio-Rad) at RT before being probed with an anti-5-LO primary antibody (66326-1-Ig Proteintech Group, Inc., Rosemont, United States) and an anti-GAPDH antibody as control (PLA0302, Merck, Darmstadt, Germany). Matching fluorescence-conjugated secondary antibodies donkey-anti-mouse (for 5-LO antibody) donkey-anti-goat (for GAPDH antibody) IRDye, LI-COR Biosciences, Bad Homburg, Germany) were used for detection with the Odyssey Infrared Imaging System (LI-COR Biosciences). For the analysis of cellular 5-LO protein expression in MV4-11 and SEM cells, 7.5 × 10^6^ cells were seeded in 15 mL RPMI (with or without 1 ng/mL TGFβ, 50 nM 1.25(OH)_2_D_3_ (VitD_3_), or the combination of both) in 10 cm dishes. After 72 h incubation cells were harvested, lysed and western blot analysis was performed as already described. The membrane was probed with an anti-5-LO primary antibody (66326-1-Ig Proteintech Group) and an anti-β-actin antibody as control (ab8229, Abcam, Cambridge, UK). Secondary antibodies used were donkey-anti-mouse for the 5-LO antibody and donkey-anti-goat for the β-actin antibody (IRDye, LI-COR Biosciences).

### Analysis of 5-LO product formation

Analysis of 5-LO activity was performed with SEM cells or MV4-11 cells after differentiation with 1 ng/mL transforming growth factor-β (TGFβ, PeproTech, Cranbury, United States), 50 nM 1,25(OH)_2_D_3_ (Cayman Chemical Company, Ann Arbor, United States) or both agents at 37°C in a humidified atmosphere with 6% CO_2_ for 72 h in cell culture flasks. To determine the 5-LO activity in intact cells, 3 × 10^6^ MV4−11 and 6 × 10^6^ SEM cells for each treatment group were harvested, and the pellet was resuspended in PBS containing 1 mg/mL glucose. 5-LO activity was stimulated by the addition of 20 µM arachidonic acid (Cayman Chemical Company, Ann Arbor, United States) and 2.5 µM calcium ionophore (A23187, Sigma Aldrich). To measure 5-LO activity in cell homogenates, 3 × 10^6^ MV4−11 and 6 × 10^6^ SEM cells were harvested, and the pellet was resuspended in PBS containing 1 mM EDTA and 1 mM ATP. The cell suspension was sonicated three times for 10 s at 10% of the maximal amplitude (Sonopuls HD 200 with Sonopuls microtip MS72). The reaction was started by the addition of 2 mM Ca^2+^ and 20 µM arachidonic acid (Cayman Chemical Company). Both, intact cells and homogenates, were incubated for 10 min at 37°C before stopping the reaction by the addition of 1 mL of ice-cold methanol (LC-MS grade, Carl Roth, Karlsruhe, Germany). Extraction of 5-LO products followed by LC-MS analysis was performed as originally described by Werz and Steinhilber, modified by Goebel and Kreiß (Werz and Steinhilber, 1996; Kreiß et al., 2022).

### siRNA-mediated gene silencing of MLL-AF4

For siRNA-mediated gene silencing of MLL-AF4 in MV4-11 and SEM cells, 0.2 × 10^6^ cells/well were seeded in 96-well cell culture plates (Greiner AG, Kremsmünster, Austria) in 200 µL Accell™ siRNA Delivery Medium (Horizon Discovery Group plc, Waterbeach, United Kingdom). Accell™ siRNA (Horizon Discovery Group plc) targeting MLL-AF4 was dissolved in siRNA buffer (Horizon Discovery Group plc) and added to the cells according to manufacturer’s protocol (final concentration of 1 µM). The following siRNA sequences were used: sense 5′-CCA​AAA​GAA​AAG​GAA​AUG​AUU-3′, antisense 5′-UCA​UUU​CCU​UUU​CUU​UUG​GUU-3´ (MV4-11) and sense 5′-CAA​AAG​AAA​AGC​AGA​CCU​AUU-3′, antisense 5′-UAG​GUC​UGC​UUU​UCU​UUU​GUU-3′ (SEM). The sequences were designed to target the cell line-specific MLL-AF4 exon-exon junctions of the two cell lines. Accell™ non-targeting control siRNA pool or Accell™ GAPD control siRNA pool cells treated analogously were used as control. MV4-11 and SEM cells were incubated with siRNA containing media for 72 h under standard culture conditions. After 72 h, cells were harvested and resuspended in PBS for further use.

## Results

### Activation of the ALOX5 promoter by MLL-AF4 is mediated by pSER, AF9-ID, CHD and CXXC domain and a five-fold tandem GC box in the ALOX5 promoter

**FIGURE 1:**
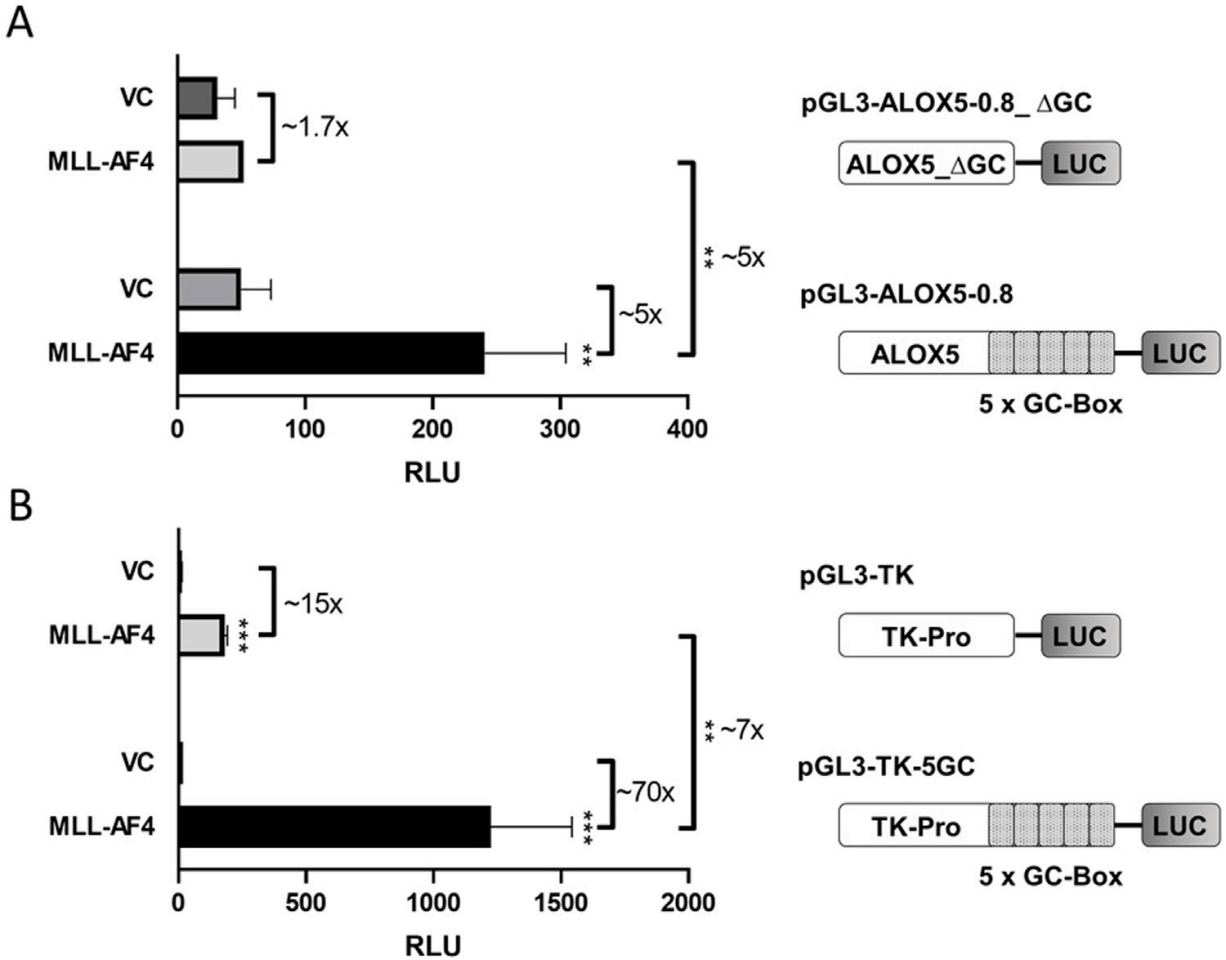
Reporter gene analysis shows GC box-dependency of MLL-AF4 activity. **(A)** HeLa cells were transfected with one of the reporter vectors pGL3-ALOX5-0.8 or pGL3-ALOX5-0.8_∆GC and with the empty expression vector control (VC) or the expression plasmid for MLL-AF4 (MLL-AF4). **(B)** HeLa cells were transfected with one of the reporter vectors pGL3-TK or pGL3-TK-5GC and with the empty expression vector control (VC) or the expression plasmid for MLL-AF4. Results are shown as relative luminescence units (RLU) normalized to the Renilla control. The values are presented as mean ± S.E.M. of three independent experiments. An unpaired *t*-test was used to determine the significance of the influence of the MLL-AF4 expression compared to VC on the according reporter construct. Asterisks indicate significant changes of MLL-AF4 compared to VC transfected cells. *p ≤ 0.05, **p ≤ 0.01, ***p ≤ 0.001.

In previous studies, it was shown by reporter gene analysis that MLL-AF4 is able to prominently induce activity of the *ALOX5* core promoter by a factor of up to 47-fold. The reporter construct employed in this analysis contained 0.8 kb of the proximal *ALOX5* promoter (plasmid pGL3-ALOX5-0.8) (Ahmad et al., 2014). In order to identify the specific sequences within this promoter region that are responsible for MLL-AF4-mediated activation, we investigated the activity of the 5-fold tandem GC box proximal to the transcriptional start site (formerly referred to as GC0-element (Schnur et al., 2007)), which is known to be crucial for basal *ALOX5* promoter activity (Schnur et al., 2007). To this end, we deleted the tandem GC element from the *ALOX5* core promoter reporter construct pGL3-ALOX5-0.8, leading to plasmid pGL3-ALOX5-0.8_∆GC. As shown in Figure 1A, coexpression of MLL-AF4 did not lead to a significant induction of the *ALOX5* promoter lacking the tandem GC box (∼1.7-fold increase), compared to the ∼5-fold upregulation when the promoter contains the GC element. In order to further investigate the activating function of the GC box, we cloned the GC element in front of the viral thymidine kinase (TK) promoter (plasmid pGL3-TK), leading to plasmid pGL3-TK-5GC. Figure 1B shows that the coexpression of MLL-AF4 as a general transcriptional activator already led to a ∼15-fold increase in reporter activity from the control plasmid pGL3-TK. An even stronger activation of ∼70-fold was observed from the plasmid carrying the tandem GC box. This approximately ∼7-fold increase in activation clearly demonstrates that the tandem repeat is the key element for MLL-AF4-mediated upregulation of *ALOX5* promoter activity.

**FIGURE 2:**
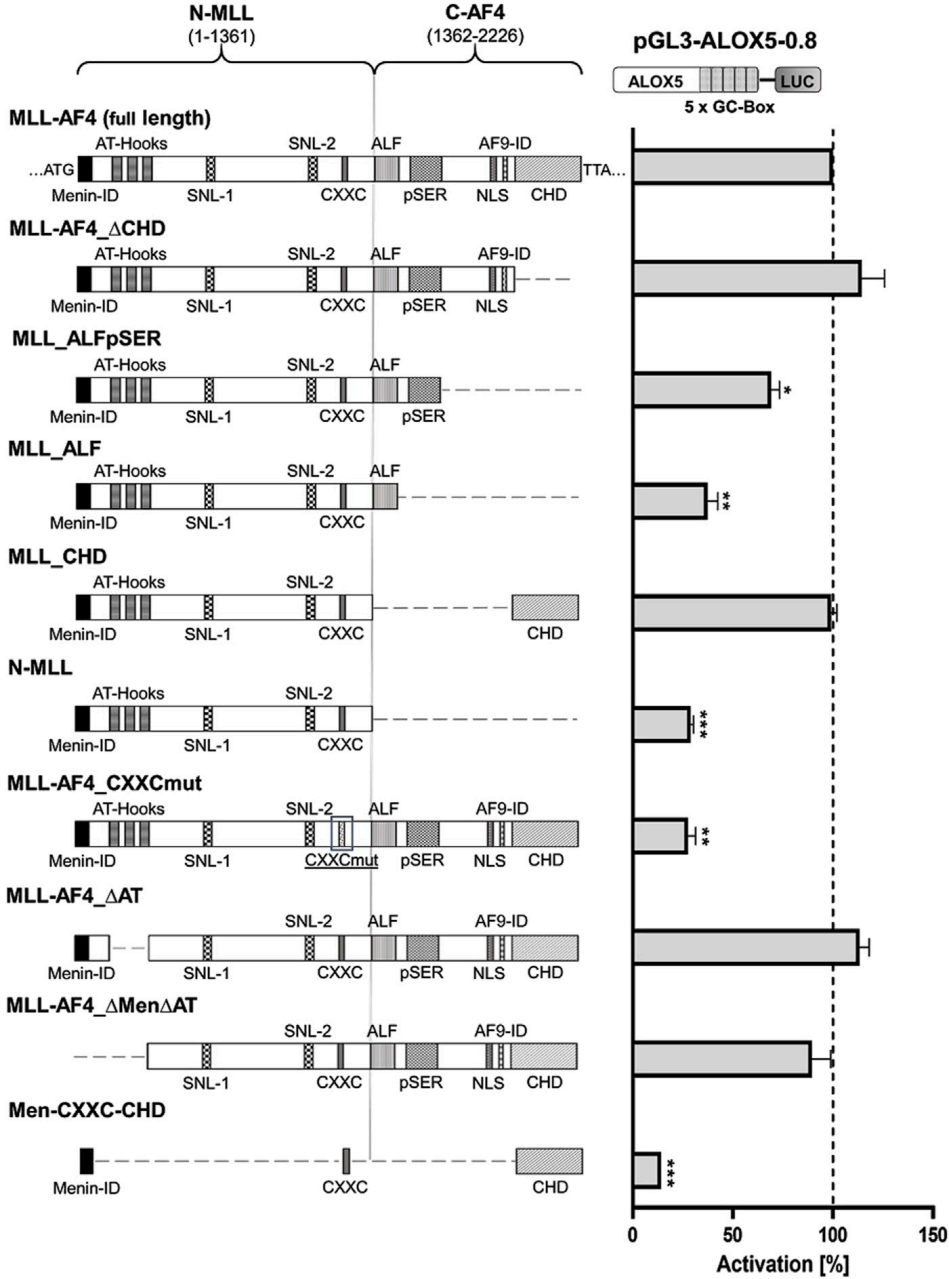
Reporter gene assay to determine GC-box-dependent transcriptional activity of MLL-AF4 mutants. HeLa cells were transfected with the full-length construct (MLL-AF4) or with one of the mutants (MLL-AF4_∆CHD, MLL_ALFpSER, MLL_ALF, MLL_CHD, N-MLL, MLL-AF4_CXXCmut, MLL-AF4_∆AT, MLL-AF4_∆Men∆AT, Men-CXXC-CHD) and a reporter plasmid containing the *ALOX5* promoter (pGL3-ALOX5-0.8). Additionally, a pRL-SV40 Renilla plasmid was cotransfected to normalize the luminescence. N-MLL: N-terminal fusion part of MLL protein, C-AF4: C-terminal fusion part of AF4, numbers represent amino acid range, Menin-ID: Menin interaction domain (El Ashkar et al., 2017; Slany, 2020), AT-Hooks: DNA binding motif (Aravind and Landsman, 1998), SNL-1, SNL-2: Speckled nuclear localization domain 1 and 2 (Yano et al., 1997), CXXC: binding motif for CpG DNA elements (MT domain) (Birke et al., 2002), ALF: family specific conserved domain (Nilson et al., 1997), pSER: Serine rich domain (Okuda et al., 2015; Siemund et al., 2022), NLS: Nuclear localization signal (Domer et al., 1993), AF9-ID: AF9 interaction domain (Bitoun et al., 2007), CHD: C-terminal homology domain (Benedikt et al., 2011; Slany, 2020). Promoter activity is displayed as % activation compared activation of pGL3-ALOX5-0.8 by full length MLL-AF4. Results (RLU) are presented as mean ± S.E.M. of three independent experiments. An unpaired *t*-test with Welch´s correction was used to determine the significance of the influence of the MLL-AF4 expression on the reporter construct compared to the mutants. *p ≤ 0.05, **p ≤ 0.01, ***p ≤ 0.001.

In a next step, we aimed to identify the regions of the multi-domain MLL-AF4 protein structure (Figure 2) that play a pivotal role in the activation of the 5-LO core promoter. To investigate this, we designed a series of expression constructs which contain either mutations or deletions of individual domains or of multi-domain segments of full-length MLL-AF4. The data indicate that several domains of MLL-AF4 play a crucial role in GC-box-dependent activation of the *ALOX5* promoter. Obviously, some domains originating from the AF4 gene locus are indispensable for MLL-AF4 effects, as shown by the strong reduction of reporter activity after deletion of the complete C-terminal part (construct N-MLL) which reduced the activity level to ∼30%. However, neither the single deletion of the CH domain (MLL-AF4_∆CHD) which is known to dimerize with wt-AF4 (Mueller et al., 2007; Benedikt et al., 2011), nor the 5′-flanking domains including the serine rich pSer domain (MLL-AF4_∆pSER) which can interact with the selectivity factor 1 (SL1) protein and the AF9-ID (MLL-AF4_∆AF9-ID) (Okuda et al., 2015; Siemund et al., 2022), result in a loss of activity (Supplementary Figure S1). The deletion of the C-terminus, including CHD and AF9-ID (MLL_ALFpSER) results in a significant reduction of activity to ∼69% (Figure 2). Finally, the additional deletion of the pSER domain (MLL_ALF) reduced the activity even further to ∼37%. Interestingly, the addition of the CH domain to the inactive N-MLL (MLL_CHD) restored full activity. In contrast, the mutation of only one amino acid within the C-terminal CXXC domain (MLL-AF4_CXXCmut) which has been described to bind hemi-methylated CpG rich DNA (Birke et al., 2002), led to a prominent reduction of the reporter signal to ∼28% residual activity compared to full-length MLL-AF4, pointing to a central role of this domain. As shown in Supplementary Figure S2 the constructs with diminished activity (N-MLL and MLL-AF4_CXXCmut) only show a ∼1.4-fold activation compared to the empty expression vector control. Regarding the MLL part of the fusion protein, we investigated the influence of a domain with AT-hooks, which was shown to be a binding motif for the DNA backbone (Aravind and Landsman, 1998), and a larger N-terminal part of MLL encompassing the AT-hooks and the N-terminal Menin binding domain (Yokoyama et al., 2005) which is known to interact with Menin-1 and Lens Epithelium-Derived Growth Factor (LEDGF) (El Ashkar et al., 2017). Both constructs, MLL-AF4_∆AT and MLL-AF4_∆Men∆AT, only led to a minor reduction in activity, which was statistically not significant (Figure 2). Based on these results, we finally attempted to design a construct of minimal size with the ability to activate the *ALOX5* promoter. We included regions of the protein that have shown to be necessary for its activity in our analysis, or are considered to be of special importance in the literature, namely, the Menin binding, CXXC and CH domains (construct Men-CXXC-CHD) (Slany, 2020). However, the Men-CXXC-CHD construct did not exhibit any significant activity on the 5-LO promoter, leading to only ∼14% residual activity. Taken together, the reporter gene data show that the CXXC domain is absolutely essential for the MLL-AF4 activity. The CHD, AF9-ID and the pSER domains are involved in mediating MLL-AF4 transcriptional elongation activity as well with redundant functions regarding *ALOX5* promoter activation.

### Various MLL-AF4 domains determine nuclear localization

**FIGURE 3:**
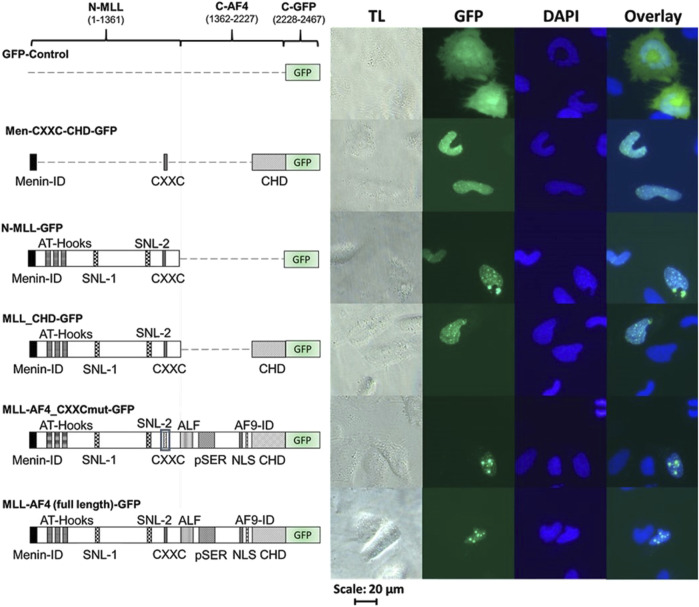
Cellular localization of MLL derivatives. Images of HeLa cells transfected with different C-terminally GFP-tagged MLL constructs or GFP protein (as control). HeLa cells were grown for 24 h and then transfected with one of the GFP-tagged constructs (GFP, Men-CXXC-CHD-GFP, N-MLL-GFP, MLL_CHD-GFP, MLL-AF4_CXXCmut-GFP, MLL-AF4-GFP) and incubated for additional 24 h. Cells were fixed with paraformaldehyde and stained with DAPI (TL = transmitted light, GFP, DAPI). Every image represents the result of one of three independent experiments.

In order to validate the correct expression and localization of the inactive constructs from Figure 2 (MLL-AF4_CXXCmut, Men-CXXC-CHD and N-MLL), fluorescence imaging was performed with the respective GFP-tagged constructs (MLL-AF4_CXXCmut-GFP, Men-CXXC-CHD-GFP, N-MLL-GFP). The constructs encoding GFP-tagged proteins with full activity in the reporter assays (MLL-AF4-GFP, MLL-CHD-GFP, Figure 2) served as positive controls. Furthermore, a plasmid expressing only GFP (GFP-Control) was used as a control for the fluorescence pattern obtained by a protein with known cytoplasmic localization such as GFP (Kitamura et al., 2015). The analysis of the microscopic images in Figure 3 revealed that stable proteins are produced from all constructs and that all proteins, with the exception of GFP alone, were localized in the nucleus. We noticed that cells transfected with N-MLL-GFP, MLL_CHD-GFP, MLL-AF4_CXXCmut-GFP and MLL-AF4-GFP exhibit a distinctly punctuated distribution of signals in the nucleus. A similar signal, however not as pronounced, was seen in some areas of the nucleus, most prominently after transfection within constructs MLL-AF4_CXXC-GFP and MLL-AF4-GFP. We can conclude that all constructs are fully expressed and exclusively localized in the nucleus.

### Heterologous expression of MLL-AF4 in 5-LO positive solid tumor cell lines HT-29 and U-2 OS does not affect ALOX5 gene expression

**FIGURE 4:**
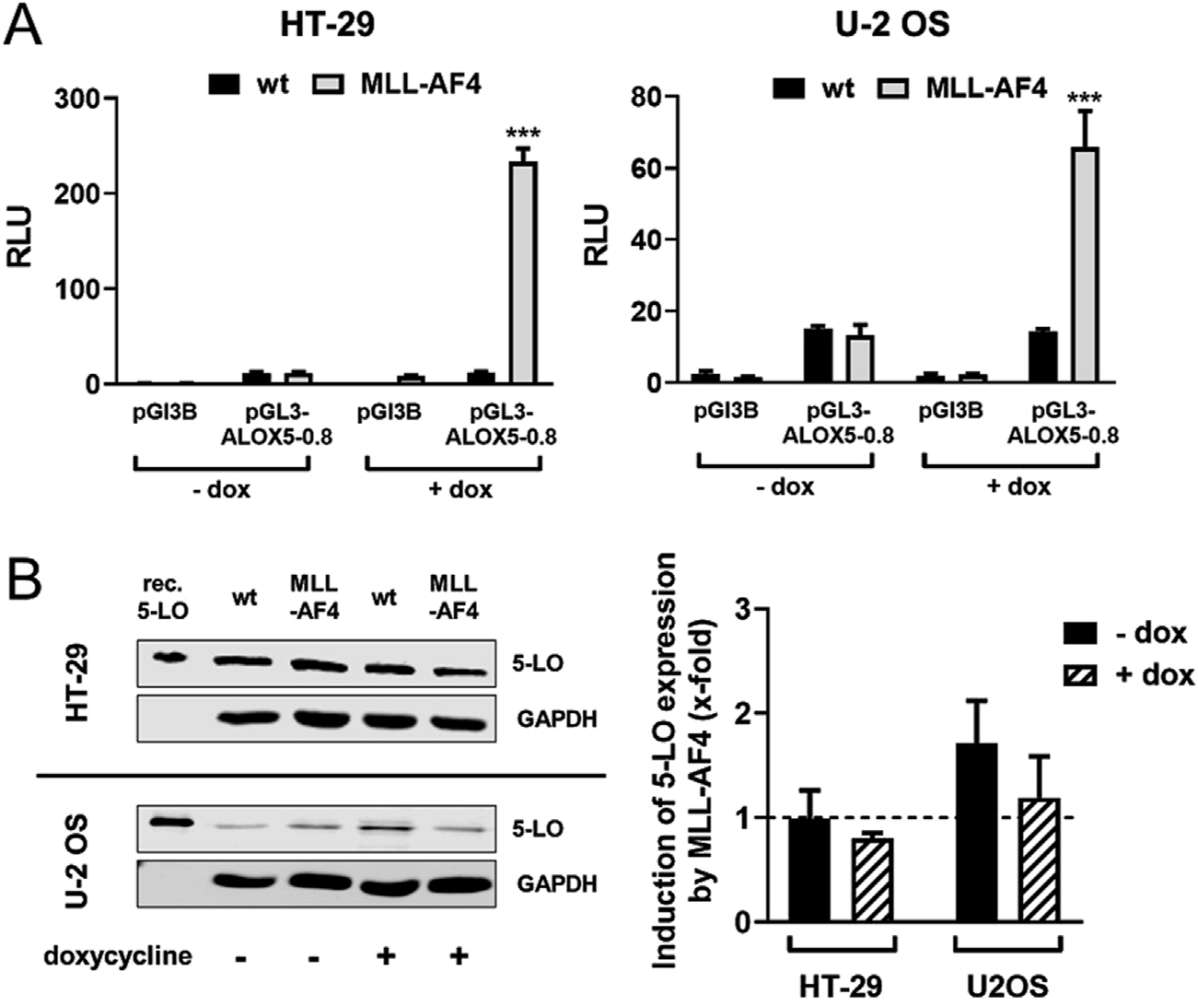
Effect of MLL-AF4 on 5-LO expression in HT-29 and U-2 OS cells. **(A)** Reporter gene analysis of HT-29 and U-2 OS wild type cells (wt) and cells stably transfected with MLL-AF4. Cells were transfected with reporter gene constructs containing the 5-LO core promoter (pGL3-ALOX5-0.8) or empty reporter vector as control (pGL3B). The activity was measured 24 h after transfection and incubation with or without doxycycline as emitted luminescence. The values were normalized to Renilla control and displayed as RLU. Results are presented as mean ± S.E.M. of three independent experiments. An unpaired *t*-test was used to determine the significance of the influence of the MLL-AF4 expressing cells compared to wild type cells. Asterisks indicate significant changes of wt cells compared to MLL-AF4 expressing cells. *p ≤ 0.05, **p ≤ 0.01, ***p ≤ 0.001. **(B)** Western blot and densitometric analysis of 5-LO expression in wild type (wt) and stably transfected and inducible MLL-AF4 positive HT-29 and U-2 OS cells with or without doxycycline (dox) treatment. Quantitative evaluation of Western blot results presented as relative 5-LO expression normalized to GAPDH and 5-LO expression in wildtype cells. Results are presented as mean ± S.E.M. of three independent experiments.

The two tumor cell lines HT-29 and U-2 OS, which are derived from a colorectal tumor and an osteosarcoma, have both been shown to prominently express 5-LO (Weisser et al., 2023). This allowed us to use these cells as model systems to analyze the effect of heterologously expressed MLL-AF4 on *ALOX5* gene expression on mRNA and protein level. For this purpose, cells were stably transfected with a doxycycline-inducible MLL-AF4 expression construct. To further validate the cell model, we checked for expression of functional MLL-AF4 protein in reporter gene assays. As can be seen from Figure 4A, induction of MLL-AF4 expression with doxycycline treatment resulted in a 60- and 220-fold increase in *ALOX5* promoter activity in the MLL-AF4 transfected cells, but not in wild type controls. No activation of reporter activity was observed with the empty vector control. These results confirm the presence of doxycycline-dependent expression of functional MLL-AF4 in these cells. To study the influence of MLL-AF4 on the activity of the genomic *ALOX5* locus, both cell lines were treated with doxycycline, or left untreated before *ALOX5* mRNA and 5-LO protein expression were analyzed by qPCR and immunoblotting, respectively. As shown in Figure 4B, induction of MLL-AF4 expression by doxycycline treatment does not affect *ALOX5* mRNA and 5-LO protein expression in these cell lines.

### siRNA-mediated knockdown of MLL-AF4 significantly represses ALOX5 gene expression in the B cell line SEM but not in the monocytic cell line MV4-11

**FIGURE 5:**
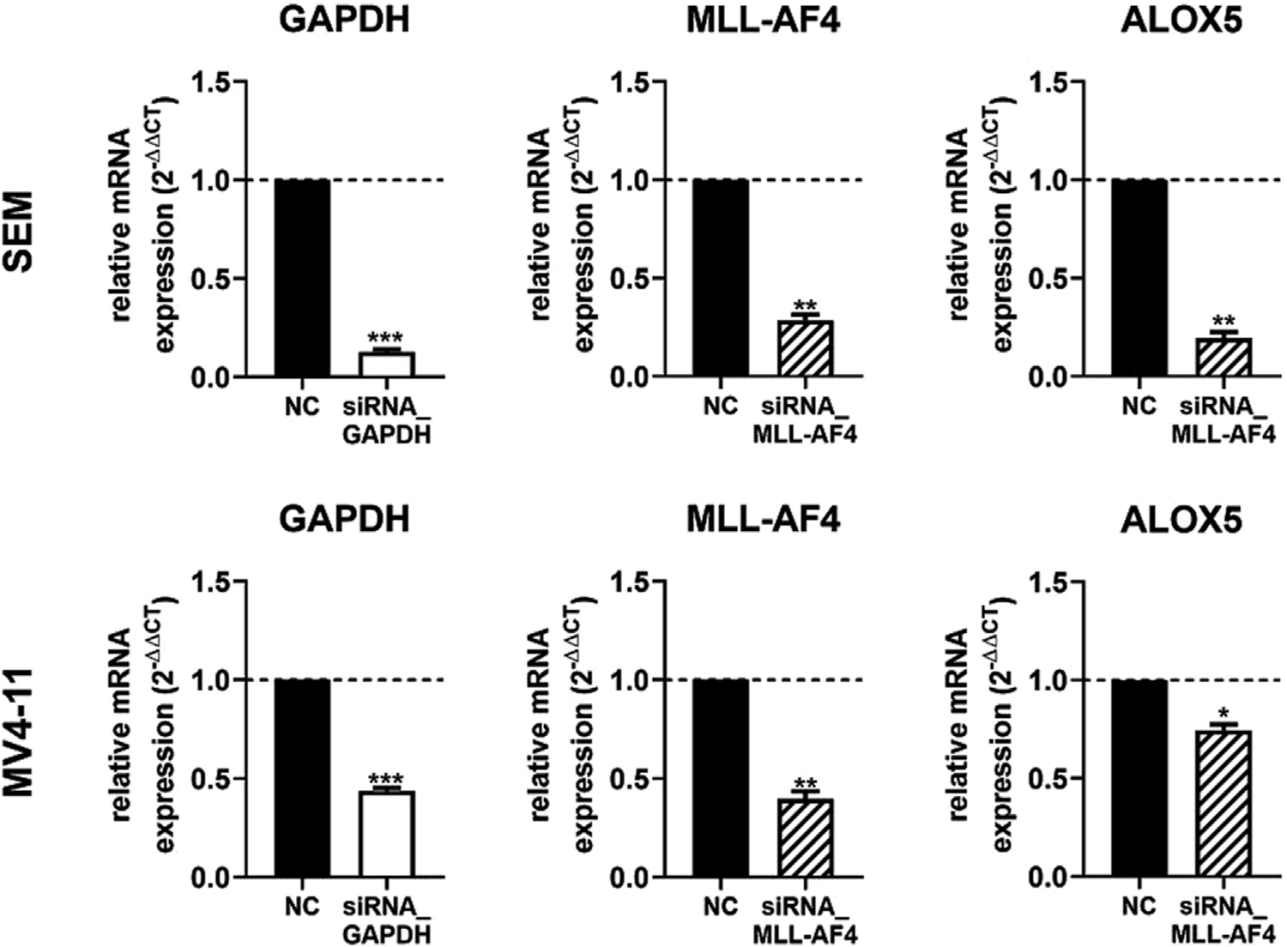
RT-qPCR analysis of 5-LO mRNA expression in siRNA-mediated MLL-AF4 knockdown cells (SEM, MV4-11). MV4-11 or SEM cells were incubated with 1 µM Accell^®^ non targeting siRNA (NC) or target siRNA (GAPDH siRNA or MLL-AF4 siRNA). Results are presented as the mean of relative mRNA expression (normalized to UBC (housekeeping gene) and compared to NC treated cells (2^−ΔΔCT^)) ± S.E.M. of three independent experiments. An unpaired *t*-test with Welch´s correction was used to determine the significance. Asterisks indicate significant changes of target siRNA treated cells to NC treated cells. *p ≤ 0.05, **p ≤ 0.01, ***p ≤ 0.001.

In a next step we wanted to investigate the effect of a MLL-AF4 knockdown in cells with native MLL-AF4 and ALOX5 expression. For this purpose, the leukemic B cell line SEM and the myelomonocytic leukemia cell line MV4-11 were used for a siRNA mediated MLL-AF4 knockdown and the 5-LO mRNA expression was investigated. Knockdown of MLL-AF4 was performed by modified, self-delivering siRNA targeting the genomic t(4,11) breakpoint junctions. In order to ensure the correct design of the siRNAs, we first confirmed the sequences of the breakpoint junctions reported in the literature for these cells (Jansen et al., 2005; Gessner et al., 2010) by qPCR (data not shown). For method validation, we used self-delivering siRNA directed against glyceraldehyde-3-phosphate dehydrogenase (GAPDH) to ensure efficient siRNA uptake in these cells, while a pool of non-targeting siRNA served as a negative control. As depicted in Figure 5, incubation of SEM and MV4-11 cells with siRNA against GAPDH resulted in a residual level of ∼13% (SEM) and ∼45% (MV4-11) of GAPDH expression, confirming successful siRNA delivery. The mRNA expression levels could be significantly reduced by the siRNAs to ∼29% (SEM) and to ∼40% (MV4-11) of non-targeting siRNA controls. However, with respect to the effects of MLL-AF4 knockdown on *ALOX5* mRNA expression, the two cell lines were differently affected. In SEM cells, *ALOX5* mRNA expression was significantly downregulated to ∼19% of the control, whereas in MV4-11 cells, although also statistically significant, the reduction of the mRNA level was only ∼74% of the control.

### ALOX5 expression by TGFβ and 1,25(OH)_2_D_3_ is induced in MV4-11 cells but not in SEM cells

**FIGURE 6:**
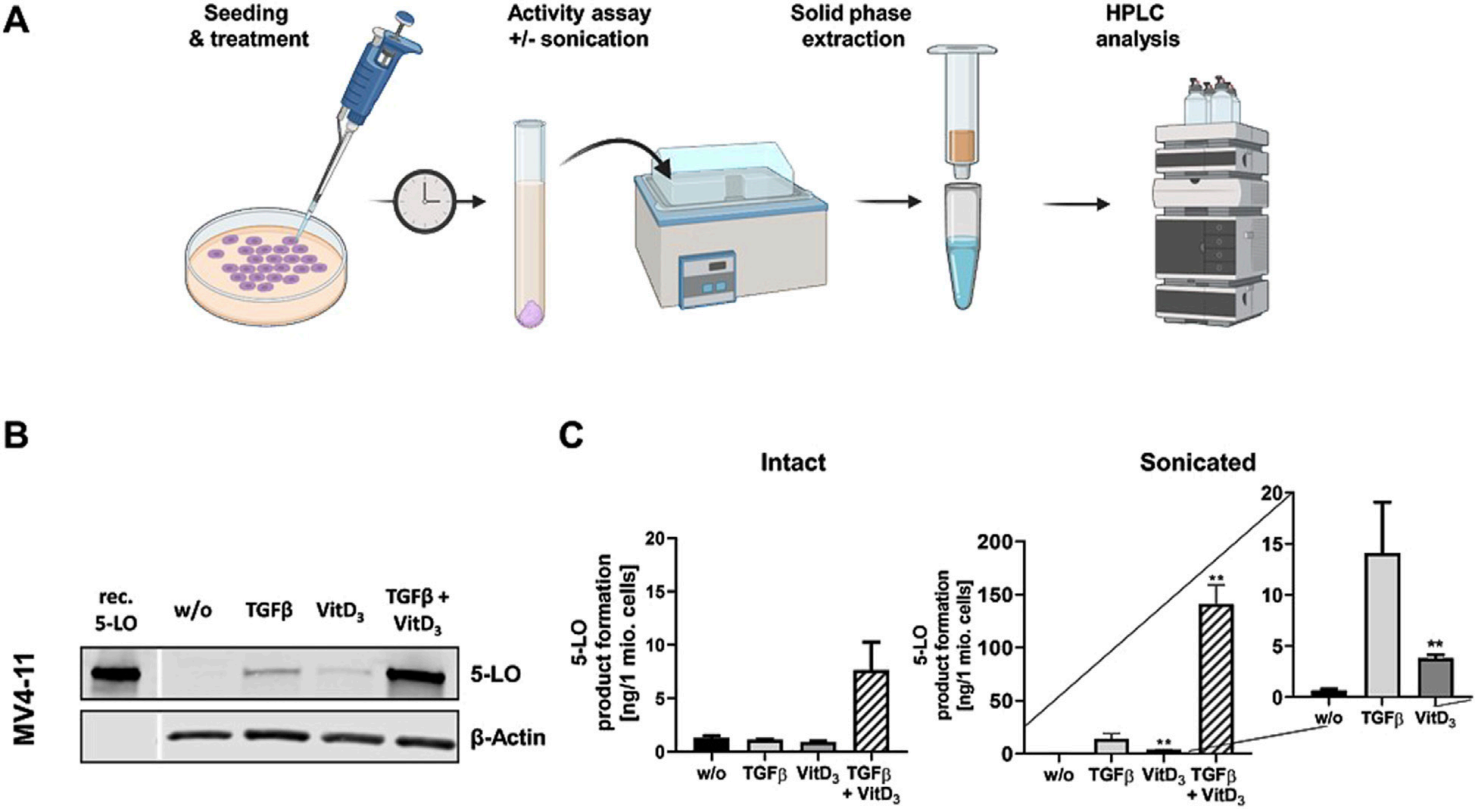
Incubation of MV4-11 cells with differentiation reagents. **(A)** Illustration of the workflow of 5-LO activity assay. **(B)** Western blot analysis of 5-LO expression in MV4-11 cells. Cells were incubated without (w/o) or with TGFβ, 1,25(OH)_2_D_3_ (VitD_3_), or the combination of both. Each blot represents the results of three independent experiments. **(C)** 5-LO product formation in MV4-11 cells after treatment with TGFβ or 1,25(OH)_2_D_3_ (VitD_3_), the combination of both or untreated cells (w/o). After 72 h 5-LO product formation was determined. Results are presented as mean ± S.E.M. of three independent experiments. Dunnet´s multiple comparison test was used to determine the significance of the influence of treated cells compared to untreated cells. Asterisks indicate significancy. *p ≤ 0.05, **p ≤ 0.01, ***p ≤ 0.001.

In conjunction with our finding that knockdown of MLL-AF4 affects *ALOX5* mRNA expression in MV4-11 and SEM cells differently (Figure 5), we analyzed whether the two cell lines display differential responsiveness of *ALOX5* gene expression and protein activity to TGFβ and 1,25(OH)_2_D_3_ that has been reported for B-cells and cells with monocytic properties (Jakobsson et al., 1992; Kreiß et al., 2022). We found that differentiation with TGFβ and 1,25(OH)_2_D_3_ induced marked morphological changes and reduced cell proliferation in MV4-11 cells, whereas SEM cells did not react to the treatment. Western blot analysis revealed a strong upregulation of 5-LO protein expression in MV4-11 cells after differentiation with TGFβ and 1,25(OH)_2_D_3_, but very low 5-LO protein expression was detected in SEM cells (Figure 6B; Supplementary Figure S3). Analysis of 5-LO activity was conducted in intact cells and cell homogenates (Figure 6A). In intact MV4-11 cells, differentiation with TGFβ and 1,25(OH)_2_D_3_ led to an upregulation of 5-LO product formation by 6-fold as compared to undifferentiated cells, whereas no 5-LO activity could be detected in differentiated and undifferentiated SEM cells. In SEM cell homogenates, we could not detect any 5-LO product formation. In contrast, 5-LO product formation in MV4-11 cell homogenates was increased ∼213-fold by treatment with TGFβ and 1,25(OH)_2_D_3_ relative to undifferentiated cells. Since the combination of TGFβ and 1,25(OH)_2_D_3_ can act synergistically on myeloid cells, whereas TGFβ and 1,25(OH)_2_D_3_ alone produce less pronounced effects, we finally tested the influence of the individual treatments. We found that differentiation with TGFβ or 1,25(OH)_2_D_3_ alone led to an increase in 5-LO activity by ∼21-fold and ∼6-fold in MV4-11 cell homogenates, respectively.

### MLL-AF9 also activates the ALOX5 promoter

**FIGURE 7:**
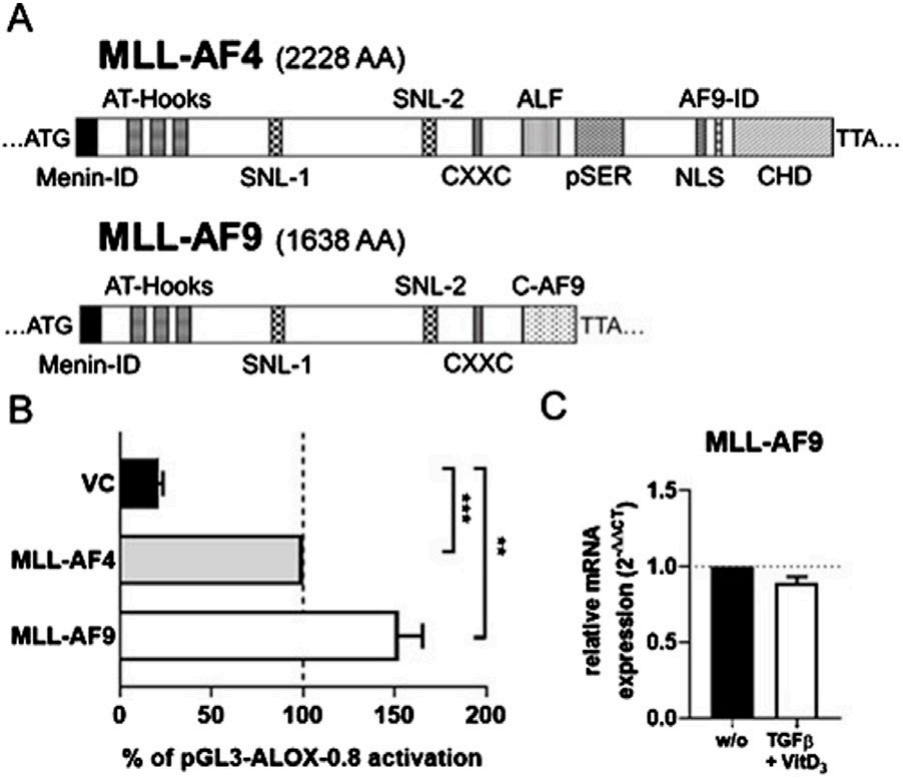
Comparison of MLL-AF4 and MLL-AF9. **(A)** MLL-AF4 and MLL-AF9 protein size in amino acids (AA). **(B)** Transcriptional activation of the *ALOX5* promoter by MLL-AF4 and MLL-AF9. HeLa cells were transfected with the expression plasmid for MLL-AF4 or MLL-AF9 and with a reporter plasmid containing the *ALOX5* promoter (pGL3-ALOX5-0.8). Promoter activity is displayed as % activation compared to activation of pGL3-ALOX5-0.8 by full length MLL-AF4, the Renilla signal was used for normalization. Results are presented as mean ± S.E.M. of three independent experiments. Welch´s *t*-test was used to determine the significance of the influence of the MLL-AF9 and VC compared to MLL-AF4 activation of pGL3-ALOX5-0.8. Asterisks indicate significant changes vs. control vector cells. *p ≤ 0.05, **p ≤ 0.01, ***p ≤ 0.001. **(C)** qPCR analysis of MLL-AF9 mRNA expression after differentiation of MonoMac-6 cells with TGFβ and 1,25(OH)_2_D_3_ for 72 h or without treatment (w/o). Results are presented as relative MLL-AF9 mRNA expression (normalized to the housekeeping gene UBC and compared to NC treated cells (2^−ΔΔCT^)).

Our finding that the chromosomal translocation product MLL-AF4 activates the *ALOX5* promoter prompted us to investigate if related MLL rearrangement proteins act in a similar fashion. To test this hypothesis, we investigated the fusion protein MLL-AF9 (Figure 7A) that is present in the monocytic cell lines MonoMac-6 and THP-1, which are frequently used model cell lines for studies on *ALOX5* expression and activity (Super et al., 1997; Pession et al., 2003). Thus, we amplified the MLL-AF9 coding sequence from MonoMac-6 cDNA and created the expression plasmid pT-MLL-AF9 which was employed in transient reporter gene assays. Interestingly, while MLL-AF4 increased 5-LO promoter activity by ∼4.7-fold compared to VC, MLL-AF9 even led to an increase of ∼7.2-fold (Figure 7B). Finally, we checked for a possible link between our findings that the fusion protein MLL-AF9 activates the *ALOX5* promoter and the long-known observation that *ALOX5* expression is strongly upregulated by TGFβ and 1.25(OH)_2_D_3_ in MonoMac-6 and THP-1 cells (Kreiß et al., 2022). However, no significant differences could be found, as shown in Figure 7C, suggesting that the strong induction of *ALOX5* expression by TGFβ and 1,25(OH)_2_D_3_ is not due to the induction of MLL rearrangement products.

## Discussion

Previous studies have shown that the fusion protein MLL-AF4 is able to induce the *ALOX5* promoter in reporter gene assays (Ahmad et al., 2014). This observation suggested a potential link between the strong leukemogenic driver protein MLL-AF4 and 5-lipoxygenase. Apart from its prominent role in inflammation (Rådmark et al., 2007; Brand et al., 2018; Kreiß et al., 2022), 5-lipoxygenase has also been associated with tumorigenesis (Kennedy and Harris, 2023; Kahnt et al., 2024) as well as with survival advantages and the aggressiveness of tumor cells (Runarsson et al., 2005; Guriec et al., 2014). As discussed, subsequently, we provide evidence on the mechanism and the cell specificity of MLL-AF4-mediated *ALOX5* gene regulation.

### The tandem GC-box of the ALOX5 promoter and the CXXC domain of MLL-AF4 are crucial for MLL-AF4-mediated ALOX5 promoter activation

The proximal *ALOX5* promoter contains a five-fold tandem consensus SP1 binding motif, which is considered the core element of the promoter responsible for basal activity (Hoshiko et al., 1990). Concomitantly, it is known that the CXXC domain of MLL-AF4 binds to hemi-methylated CpG-rich elements (Birke et al., 2002), pointing to an interaction between MLL-AF4 and the *ALOX5* promoter via GC boxes. In line with this, we could show by reporter analysis that the five-fold tandem GC-box into the viral thymidine kinase promoter renders this promoter inducible by MLL-AF4. Second, we found that deletion of the GC-box from the *ALOX5* promoter sequence significantly decreases its responsiveness to MLL-AF4. Conversely, we show through targeted mutation of the CXXC domain that MLL-AF4 activation of the *ALOX5* promoter depends on this element, as CXXC mutation dramatically reduces the induction of reporter gene activity by the CXXC mutant. This suggests a crucial role of the GC-boxes and the CXXC domain. It is noteworthy that the tandem GC-box which serves as the primary binding motif for MLL-AF4 is subject to naturally occurring polymorphisms. In a study, 6% of asthma patients exhibited mutations within this GC-box arrangement, leading to an unresponsiveness to treatment with 5-LO targeting medications like zileuton. Thus, it would be interesting, whether alterations in the GC box of *ALOX5* is of relevance in the context of leukemias carrying MLL-containing fusion proteins such as MLL-AF4 (Drazen et al., 1999).

### pSER, AF9-ID and CH domains of MLL-AF4 redundantly mediate ALOX5 promoter activation

**FIGURE 8:**
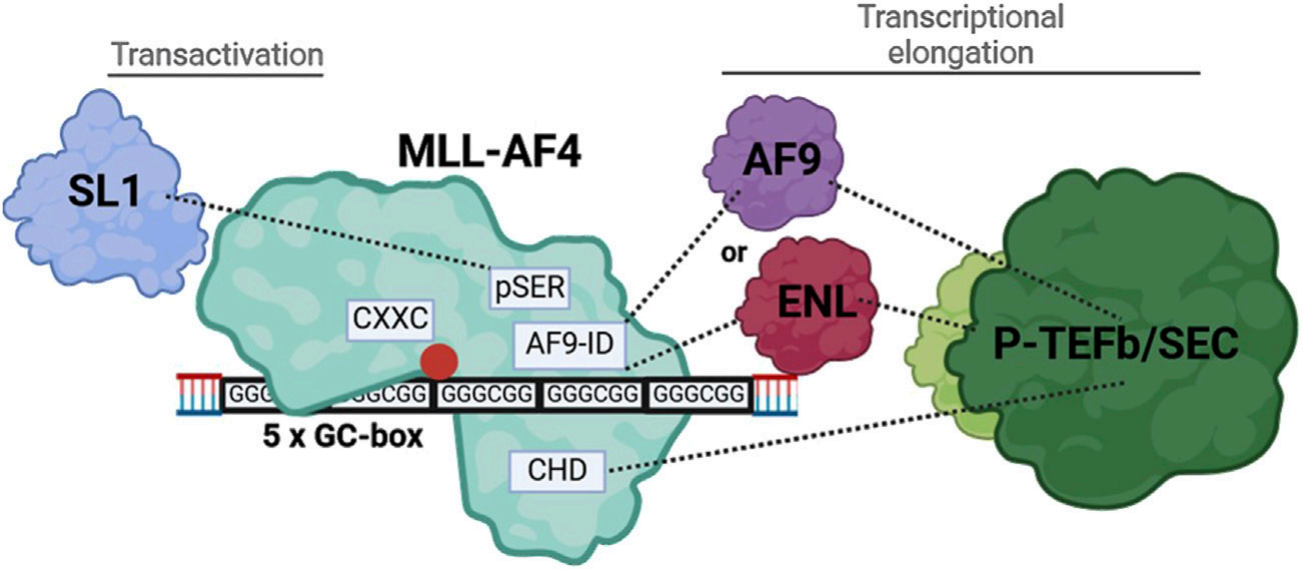
Illustration of the interaction of MLL-AF4 with the tandem GC-box within the *ALOX5* promoter and the recruitment of interaction partners, resulting in increased gene expression.

MLL-AF4, as a prominent leukemogenic product of MLL-r (MLL gene rearrangements), contains a multitude of protein domains whose functions are not yet fully understood (Lavau et al., 1997). We found that in addition to the CXXC domain, distinct domains of the AF4-part of MLL-AF4 are essential for the *ALOX5* promoter activation (see below) but that the deletion of the Menin binding domain and thus the interaction with LEDGF is of minor importance and that the DNA binding AT-hooks do not play a significant role in *ALOX5* promoter activation (Figure 2) (Yokoyama et al., 2005; El Ashkar et al., 2017). In contrast, complete deletion of the AF4 fragment (construct N-MLL) strongly diminished the transactivation potency of the mutants to levels comparable with the CXXC mutant, which shows that at least one of the redundantly acting AF4 segments is necessary for the activity of the fusion protein. The deletion analysis of the AF4 part suggests that the pSer, AF9-ID and CH domains have redundant functions in 5-LO promoter activation (Figure 2). In addition, a deletion of both CHD and AF9-ID (construct MLL_ALFpSER) results in a moderately active fusion protein that is only ∼69% active compared to the full-length construct. This suggests that either the interaction with ENL or AF9 via AF9-ID or the interaction with the AF4 wild-type complex via CHD may be sufficient to recruit the P-TEFb/SEC (super elongation complex) and initiate transcriptional elongation of promoter-proximal arrested RNA polymerase (POL A) via conversion into elongating RNA polymerase (POL E) (Mueller et al., 2009; Luo et al., 2012; Slany, 2020). This would explain why there is no simultaneous requirement for both domains to interact with their protein partners, provided that there is at least one interaction of the MLL-AF4 fusion protein with P-TEFb/SEC (Lin et al., 2010; He et al., 2011; Luo et al., 2012; Fujinaga et al., 2023). The remaining activity of MLL_ALFpSER could be explained by the fact, that the pSER domain can still fulfil a transactivation function via recruitment of the selective factor 1 complex (Okuda et al., 2015; Siemund et al., 2022). Our findings are summarized in Figure 8. Finally, to find a minimal functional MLL-AF4 mutant, we designed a construct (Men-CXXC-CHD), containing the putative essential domains based on our reporter gene assays. Surprisingly, the construct remained inactive for an as yet unknown reason.

### Nuclear localization of mutated MLL-AF4 constructs

For the inactive MLL-AF4 mutants (N-MLL, MLL-AF4_CXXCmut, Men-CXXC-CHD) we found that all constructs are expressed and located in the nucleus so that the lack of activity is not due to a failure of protein expression and a lacking import into the nucleus, rather to a loss of function (Figure 3). The observation that MLL-AF4-GFP and MLL-AF4_CXXCmut-GFP exhibit a highly punctuated distribution within the nucleus is in agreement with findings of previously published studies on the N-MLL protein, where it was suggested that this punctuated pattern is likely to be associated with wt-MLL binding DNA (Yano et al., 1997) and a formation of transcriptional, highly active micro compartments (Rasouli et al., 2024). However, even the construct with a mutated CXXC domain (MLL-AF4_CXXCmut-GFP), exhibits this speckled nuclear distribution, although the mutated CXXC domain should no longer be able to bind to DNA. This could indicate that the DNA binding is transmitted through an additional protein region (e.g., AT-hooks) which is not able to substitute for the CXXC domain binding towards GC boxes but can mediate interaction with DNA (Reeves and Nissen, 1990; Aravind and Landsman, 1998). Furthermore, it is worth mentioning that even the smallest construct (Men-CXXC-CHD) is located in the nucleus, even though it does not contain nuclear localization sequences. This could be a hint for a shuttling mechanism which could be transmitted through the CH domain, working as an interaction platform for ENL and with this for AF9. It is known that both proteins, ENL and AF9, are located in the nucleus and could shuttle Men-CXXC-CHD to the same destination (Rubnitz et al., 1994; Erfurth et al., 2004; Kabra and Bushweller, 2022).

### Regulation of ALOX5 gene in solid tumor cells is not affected by MLL-AF4 co-expression

The knock-in and the expression of the MLL-AF4 fusion gene into the colorectal cancer cell line HT-29 and the osteosarcoma cell line U-2 OS did not result in a significant change in the expression of the 5-LO (Figure 4B). Despite demonstrating that MLL-AF4 is expressed and active following the induction with doxycycline and the followed induction of the *ALOX5* reporter system, we did not see any change in 5-LO protein levels, when the cells express MLL-AF4 (Figure 4A). This indicates that the native *ALOX5* promoter is regulated differently in these solid tumor cell lines, compared to the transiently transfected pGL3-ALOX5-0.8 reporter construct.

### Differential regulation of ALOX5 expression by MLL-AF4 as well as TGFβ and 1,25(OH)₂D₃ in SEM and MV4-11 cells

So far, the mechanisms involved in 5-LO pathway activation in lymphoid and myeloid leukemia is very limited. It was reported that a loss of the ALOX5 gene prevents the outbreak of leukemia in a mouse model (Chen et al., 2009). Even though this study needs independent reproduction it is clear evidence, that 5-LO could play a major role in the development and progression of malignant blood diseases. To get a better insight into the mechanisms behind the ALOX5 activation, we used SEM and MV4-11 cells which both carry the chromosomal translocation t(4;11)(q21;q23), resulting in the expression of two reciprocal fusion proteins, MLL-AF4 and AF4-MLL, and performed MLL-AF4 knockdown experiments. It is known that malignant B-cells can over express 5-LO, but so far this regulation does not lead to increased 5-LO metabolite formation suggesting that 5-LO might have non-canonical functions in these cell lines (Jakobsson et al., 1992; Kahnt et al., 2024). However, 5-LO mRNA and protein expression in SEM cells is not upregulated (Karlsson et al., 2021; Proteinatlas, 2024). Interestingly, *ALOX5* gene expression in SEM cells is significantly downregulated by the MLL-AF4 knockdown (Figure 5 SEM). However, we were not able to detect MLL-AF4 or 5-LO via Western blotting due to low expression levels. Interestingly, another study in 1995 encompassing eight samples of B-ALL patients, showed that only four of the tested cells expressed 5-LO (Feltenmark et al., 1995). In contrast to SEM cells, the knockdown of MLL-AF4 only slightly affected *ALOX5* mRNA expression in MV4-11 cells in our study (Figure 5, MV4-11), indicating that the *ALOX5* regulation is different in both cell lines. This is supported by the observation that 5-LO expression and activity is strongly induced by TGFβ and 1,25(OH)₂D₃ in MV4-11 cells but not in SEM cells (Figure 6). The elevated formation of 5-LO pathway metabolites is an interesting finding, as it was already published that the expression and the formation of 5-LO products can contribute to an inflammatory environment that promotes malignant progression and chemotherapeutic resistance in myeloid leukemia (Runarsson et al., 2007; Vincent et al., 2008; Stranahan et al., 2022). We could previously show that induction of 5-LO gene expression in myeloid cells by TGFβ and 1,25(OH)₂D₃ is mainly due to transcript elongation (Sorg et al., 2006; Rådmark et al., 2007; Stoffers et al., 2010; Ahmad et al., 2015). Our data on the MLL-AF4 fusion protein and its dependence on the tandem GC box in the *ALOX5* promoter as well as the CXXC domain suggests that its activity is related to transcriptional initiation. Interestingly, previous studies showed that the reciprocal fusion protein of MLL-AF4, AF4-MLL (N-terminal AF4 fused with C-terminal MLL) mediates the responsiveness of the ALOX5 gene to induction by TGFβ and 1,25(OH)₂D₃ which is associated with regulatory elements in the distal parts of the *ALOX5* gene and related to transcriptional elongation (Ahmad et al., 2015). Thus, the *ALOX5* gene in SEM cells appears to be more promoter driven by MLL-AF4 whereas in MV4-11 cells induction of transcriptional elongation by TGFβ and 1,25(OH)₂D₃ mainly drives *ALOX5* expression. Whereas 5-LO expression and activity is high in differentiated myeloid cells and in the majority of B cell lines, the low 5-LO expression in SEM cells and the lack of cellular activity could point to a role of 5-LO as transcriptional regulator and regulator of cell proliferation in this cell line (Jakobsson et al., 1995; Mahshid et al., 2009; Kreiß et al., 2022; Claesson et al., 2024).

### MLL-AF9 and MLL-AF4 similarly activate the ALOX5 promoter

MLL-AF9 is the translocation product of the KMT2A gene and the MLLT3 Super Elongation Complex Subunit gene (MLLT3). This fusion occurs much more prominent in acute myeloid leukemias (Meyer et al., 2013). The resulting fusion protein MLL-AF9 contains the same N-terminal MLL domains as MLL-AF4 but has a different C-terminus. The finding that MLL-AF9 induces the *ALOX5* even stronger compared to MLL-AF4 is of high interest taking the fact that the C-terminal AF9 portion in MLL-AF9 is much smaller than C-terminal AF4 in MLL-AF4 which provides a much smaller interaction surface for other proteins of the P-TEFb/SEC (Figure 7A). It is known that MLL-AF9 interacts with members of the super elongation complex such as wt-AF4 and PAF1 via its C-terminal ANC1 homology and YEATS domain (AHD) (Pession et al., 2003; He et al., 2011). Thus, a common mechanism of MLL-AF4 and MLL-AF9 could be the recruitment of the AF4 super elongation complex (SEC) via the AF9-ID or CHD portion of the protein (Steinhilber and Marschalek, 2018). This finding is in line with our observation that only one C-terminal interactive domain in MLL-AF4 is needed to recruit the P-TEFb/SEC elongation complex, pointing towards a similarity between the activation mechanism of MLL-AF4 and MLL-AF9. AML cells, such as MonoMac-6 and THP-1, carrying the MLL-AF9 translocation, show strong *ALOX5* induction by TGFβ and 1,25(OH)_2_D_3_ (Brungs et al., 1995; Kreiß et al., 2022), similar to our findings with MV4-11 cells (Figure 6C). However, we did not observe significant changes in MLL-AF9 expression suggesting that the effects of TGFβ and 1,25(OH)_2_D are not due to induction of MLL-AF9 but are related to different, yet unknown mechanisms. Of note, it will be interesting to study ALOX5 expression in freshly isolated AML cells carrying MLL translocations.

Taken together, we could show that MLL-AF4 and MLL-AF9 strongly activate the *ALOX5* promoter in B-lymphocytic cells and that the MLL-AF4 effects are mediated by the tandem GC box in the *ALOX5* promoter. Furthermore, we could identify several AF4 domains known to bind the super elongation complex that are essential for the induction of *ALOX5* promoter activity.

## Data Availability

The raw data supporting the conclusions of this article will be made available by the authors, without undue reservation.
