# Supplementary material for: MLL-AF4 upregulates 5-lipoxygenase expression in t(4;11) leukemia cells via the ALOX5 core promoter
Source: Front Pharmacol. 2025 Jan 14;15:1520507. doi: 10.3389/fphar.2024.1520507 (PMC11772344; doi:10.3389/fphar.2024.1520507)
Supplement: Supplementary file 1 [file DataSheet1.pdf]

Supplementary information

MLL-AF4 upregulates ALOX5 expression in t(4;11) leukemia cells via the ALOX5 core promoter

Marius Hyprath[1], Maximilian Molitor[1], Ilona Schweighöfer[1], Rolf Marschalek[2] and Dieter Steinhilber[1]

[1]Institute of Pharmaceutical Chemistry, [2]Institute of Pharmaceutical Biology, Goethe University, Max-von-Laue-Str. 9, D-60438 Frankfurt, Germany.

Figure S1

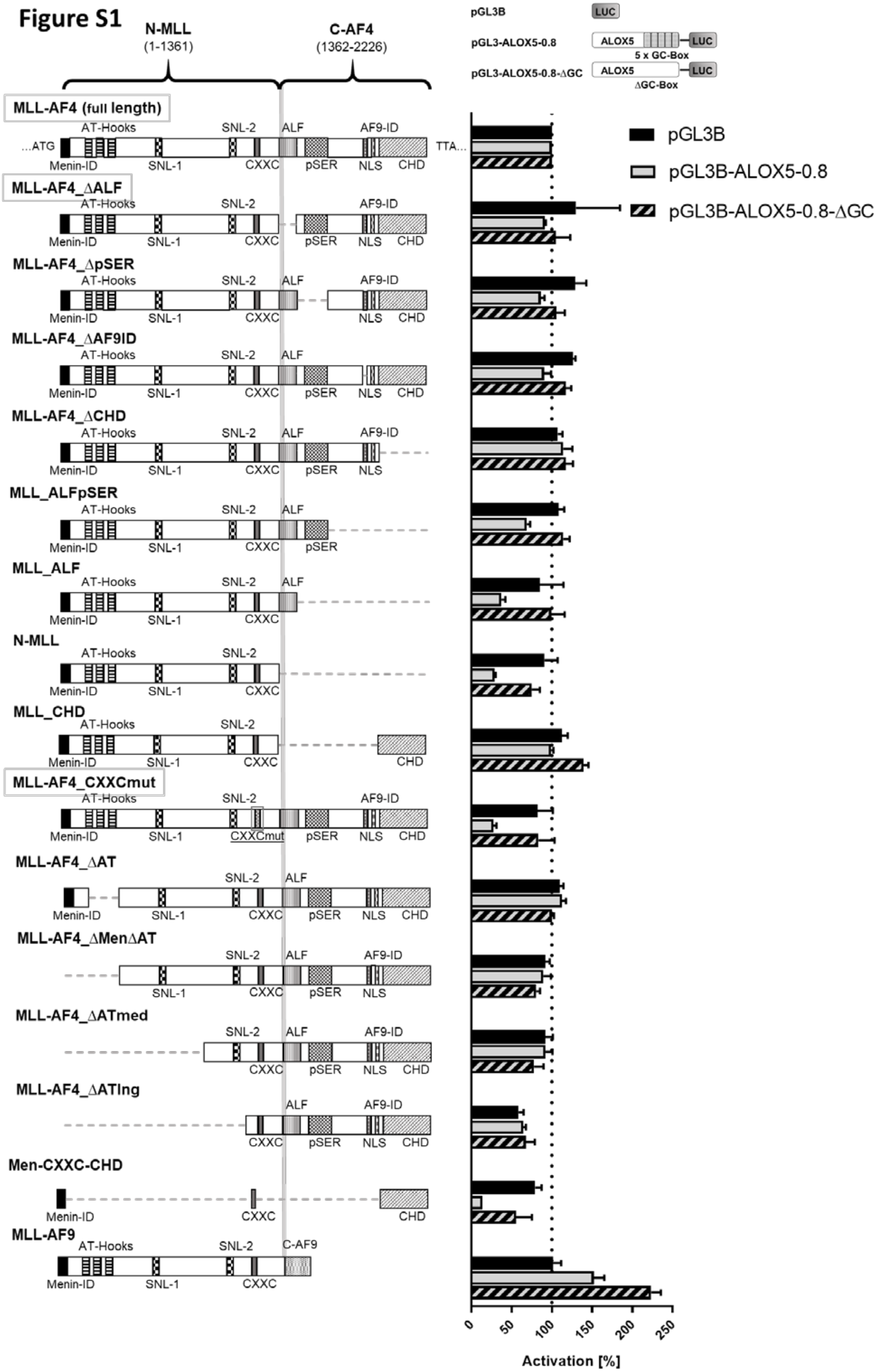

N-MLL
(1-1361)
C-AF4
(1362-2226)
pGL3B
LUC
pGL3-ALOX5-0.8
ALOX5
5 x GC-Box
pGL3-ALOX5-0.8-ΔGC
ΔGC-Box
MLL-AF4 (full length)
AT-Hooks
SNL-2
ALF
AF9-ID
...ATG
TTA...
Menin-ID
SNL-1
CXXC
pSER
NLS
CHD
MLL-AF4_ΔALF
MLL-AF4_ΔpSER
MLL-AF4_ΔAF9ID
MLL-AF4_ΔCHD
MLL_ALFpSER
MLL_ALF
N-MLL
MLL_CHD
MLL-AF4_CXXCmut
CXXCmut
MLL-AF4_ΔAT
MLL-AF4_ΔMenΔAT
MLL-AF4_ΔATmed
MLL-AF4_ΔATlng
Men-CXXC-CHD
MLL-AF9
C-AF9
pGL3B
pGL3B-ALOX5-0.8
pGL3B-ALOX5-0.8-ΔGC
0
50
100
150
200
250
Activation [%]

Figure S1. Effect of different mutants of the full-length construct MLL-AF4 on the empty report control (pGL3B), the 5-LO core promoter (pGL3-ALOX5-0.8) and the 5-LO core promoter lacking the tandem GC-box (pGL3-ALOX5-0.8-ΔGC). HeLa cells were transiently transfected with the full-length construct (MLL-AF4) or with one of the mutants MLL-AF4_ΔALF (AA 1-1362, AA 1455-2226 (ΔAA 1363-1454)), MLL-AF4_ΔpSER (AA 1-1406, AA 1576-2226 (ΔAA 1407-1575)), MLL-AF4_ΔAF9ID (AA 1-1776, AA 1791-2226 (ΔAA 1777-1790)), MLL-AF4_ΔCHD (AA 1-1869 (ΔAA 1870-2226)), MLL_ALFpSERsh (AA 1-1537 (ΔAA 1538-2226)), MLL_ALF (AA 1-1455 (ΔAA 1456-2226)), N-MLL (AA 1-1362 (ΔAA 1363-2226)), MLL_CHD (AA 1-1362, 1871-2226 (ΔAA 1363-1870)), MLL-AF4_CXXCmut (AA 1188 C→D), MLL-AF4_ΔAT (AA 1-169, AA 309-2226 (ΔAA 170-308)), MLL-AF4_ΔMenΔAT (AA 1-1, AA 309-2226 (ΔAA 2-308)), MLL-AF4_ΔATmed (AA 1-169, AA 908-2226 (ΔAA 170-907)), MLL-AF4_ΔATlng (AA 1-169, AA 1147-2226 (ΔAA 170-1146)), Men-CXXC-CHD (AA 1-18, AA 1148-1203, AA 1871-2226 (ΔAA 19-1147, ΔAA 1204-1870)) together with pGL3B, pGL3-ALOX5-0.8 or pGL3-ALOX5-0.8-ΔGC. The activity was measured 24 h hours after transfection as emitted luminescence. The values were normalized against Renilla luminescence and were displayed as % activation compared to MLL-AF4 on pGL3B, pGL3-ALOX5-0.8 or pGL3-ALOX5-0.8-ΔGC. Results are presented as mean ± S.E.M. of three independent experiments.

Figure S2

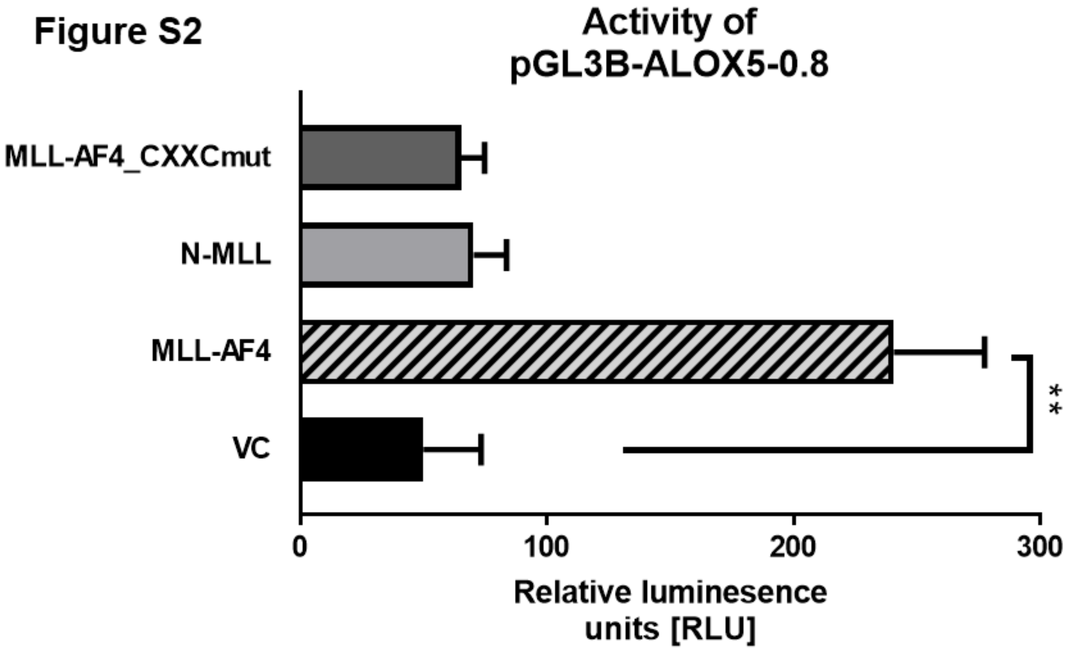

Figure S2. Comparison of reporter gene activity of full-length MLL-AF4, MLL-AF4_CXXCmut and N-MLL with the empty expression vector control (VC) on the ALOX5 core promoter (pGL3B-ALOX5-0.8). HeLa cells were transiently transfected with the corresponding expression plasmids or VC together with pGL3-ALOX5-0.8. Additionally, a pRL-SV40 Renilla plasmid was cotransfected to normalize the luminescence. The activity was measured 24 h hours after transfection as emitted luminescence. The values were normalized against Renilla luminescence and were displayed as relative luminescence units. Results are presented as mean ± S.E.M. of three independent experiments. An unpaired t-test was used to determine the significance of the influence of the expression plasmids compared to VC. $^*P \leq 0.05$, $^{**}P \leq 0.01$, $^{***}P \leq 0.001$.

Figure S3

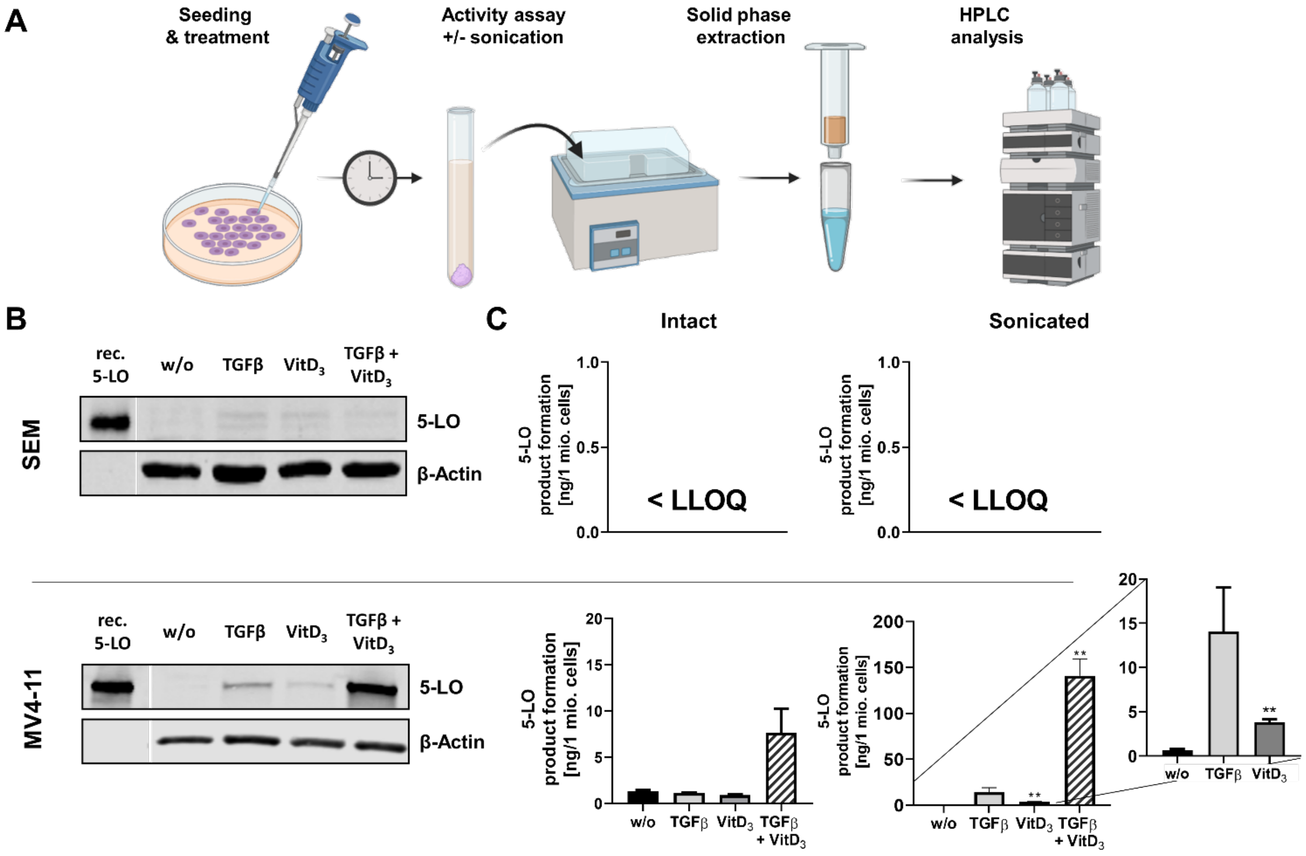

Figure S3. Incubation of MV4-11 and SEM cells with differentiation reagents A) Illustration of the workflow of 5-LO activity assay. **B)** Western blot analysis of 5-LO expression in SEM and MV4-11 cells. Cells were incubated without (w/o) or with TGFβ, 1,25(OH)$_2$D$_3$ (VitD$_3$), or the combination of both. Each blot represents the results of three independent experiments. **C)** 5-LO product formation in SEM (top) and MV4-11 cells (bottom) after treatment with TGFβ or 1,25(OH)$_2$D$_3$ (VitD$_3$), the combination of both or untreated cells (w/o). MV4-11 cells and SEM cells were cultured without or in presence of TGFβ, 1,25(OH)$_2$D$_3$ (VitD$_3$), or the combination of both. After 72 h 5-LO product formation was determined. LLOQ. = Lower limit of quantification. Results are presented as mean ± S.E.M. of three independent experiments. Dunnet´s multiple comparison test was used to determine the significance of the influence of treated cells compared to untreated cells. Asterisks indicate significancy. *P ≤ 0.05, **P ≤ 0.01, ***P ≤ 0.001.

Table S1. List of used cloning oligonucleotides (forward and reverse), templates and restriction enzymes (RE) for PCR, restriction digest and NEBuilder HiFi DNA Assembly (New England Biolabs). The plasmids pTarget, MLL-AF4 and EGFP were obtained from different sources (see materials and methods).

pTarget	Template	Forward	Reverse	RE for backbone
Primer for fragment A	-	-	-	-
Primer for fragment B	-	-	-	-
**MLL-AF4**	**Template**	**Forward**	**Reverse**	**RE for backbone**
Primer for fragment A	-	-	-	-
Primer for fragment B	-	-	-	-
**MLL-AF4_∆ALF**	**Template**	**Forward**	**Reverse**	**RE for backbone**
Primer for fragment A	MLL-AF4	GTGCGAGGACCCCGGATTAAA	GAGGGAGGCTTCTCTGGGGTCTTTTCTTTTGGTTTTTGTTTTACAGGGATAC	pciI
Primer for fragment B	MLL-AF4	ACCCCAGAGAAGCCTCCCTCC	GTCTATCAGGGCGATGGCCCAC	draIII
**MLL-AF4_∆pSER**	**Template**	**Forward**	**Reverse**	**RE for backbone**
Primer for fragment A	MLL-AF4	GTGCGAGGACCCCGGATTAAA	CTGGAGCTTTTAGGGGGAGG CTTTGTAGGGAAAGGAAACTTG	pciI
Primer for fragment B	MLL-AF4	CCTCCCCCTAAAAGCTCCAGC	CTATCAGGGCGATGGCCCAC	draIII
**MLL-AF4_∆AF9ID**	**Template**	**Forward**	**Reverse**	**RE for backbone**
Primer for fragment A	MLL-AF4	GCTTTGTGGGGGAGGAGTGTC	CTCCCCTTCCCGGGAGGCTG GCTTTGTGGGGGAGGAGTGTC	pciI
Primer for fragment B	MLL-AF4	CAGCCTCCCGGGAAGGGGAG	GTCTATCAGGGCGATGGCCCAC	draIII

MLL-AF4_ΔCHD	Template	Forward	Reverse	RE for backbone
Primer for fragment A	MLL-AF4	GTGCGAGGACCCCG GATTAAA	AATCAACCTGGGGC TCCATTA CCTGGAGGGCTTTG TTTTAGAAG	pciI
Primer for fragment B	MLL-AF4	TAATGGAGCCCCAG GTTGATTCAATG	GTCTATCAGGGCGA TGGCCCAC	draIII
**N-MLL**	**Template**	**Forward**	**Reverse**	**RE for backbone**
Primer for fragment A	MLL-AF4	GTGCGAGGACCCCG GATTAAA	GAATCAACCTGGGG CTCCATTACTTTTCT TTTGGTTTTTGTTTTA CAGGG	pciI
Primer for fragment B	MLL-AF4	TAATGGAGCCCCAG GTTGATTCAATG	CTATCAGGGCGATG GCCCAC	draIII
**MLL_ALF**	**Template**	**Forward**	**Reverse**	**RE for backbone**
Primer for fragment A	MLL-AF4	CCTTAATGGAGCCC CAGG	GGTTTGTTCACTGTC ACTGTC	pciI
Primer for fragment B	MLL-AF4	GTGCGAGGACCCCG GATTAAA	GAATCAACCTGGGG CTCCATTA TTGTTCACTGTCACT GTCCTCAC	draIII
**MLL_ALFpSERsh**	**Template**	**Forward**	**Reverse**	**RE for backbone**
Primer for fragment A	MLL-AF4	GTGCGAGGACCCCG GATTAAA	ATCAACCTGGGGCT CCATTA TTTGGTCAGCCAGTT GTCCA	pciI
Primer for fragment B	MLL-AF4	TAATGGAGCCCCAG GTTGATTCAATG	CTATCAGGGCGATG GCCCAC	draIII
**MLL-AF4_ΔATlng**	**Template**	**Forward**	**Reverse**	**RE for backbone**
Primer for fragment A	MLL-AF4	GCAGCCCGGGGGAT CCGG	CTCGATCGACGTCC TTTCTT TTTAACTGAAGGAGA CCTTGTGGGACTTC	Acc65I
Primer for fragment B	MLL-AF4	AAGAAAGGACGTCG ATCGAGGC	CCTGGCTCCCTTTCC CCC	SbfI

Men-CXXC-CHD	Template	Forward	Reverse	RE for backbone
Primer for fragment A	MLL_CHD	GCCCCCGGTGGTCC CGG	CCCTCCTCACAGTC CTCAAAGAAGGAAAT G	-
Primer for fragment B	MLL_CHD	GACCCGGGACCACC GGGGGC AAAGGACGTCGATC GAGGCG	TTTGAGGACTGTGA GGAGGG TTTGGAAGGCATCCA TTGTAGATTCTG	-

MLL-AF4_CXXCmut	Template	Forward	Reverse	RE for backbone
Primer for fragment A	MLL-AF4	GTGCGAGGACCCC GGATTAAA	CTGCTTCTTTATATTG CGACCACC	pciI
Primer for fragment B	MLL-AF4	GCAAGATGAGAAA ATGTCAGAATC	CTACTGCTTTTCTTTG GGGC	BamHI
Mutated oligo insert	GTCGCAATATAAAGAAGCAG**GA**CTGCAAGATGAGAAAATGTCAG			-

MLL-AF9	Template	Forward	Reverse	RE for backbone
Primer for fragment A	MLL-AF4	GTGCGAGGACCC CGGATTAAA	TTCTGTTTGCTCTGCT CTGGACC	pciI
Primer for fragment B	MLL-AF4	GGAAACATCTGGA ACATCCTGA TGGAGCCCCAGG TTGATTCAATG	CTATCAGGGCGATGG CCCAC	draIII
Primer for fragment C	cDNA from MONO-MAC-6 cells	TCCAGAGCAGAG CAAACAGAA	TCAGGATGTTCCAGAT GTTTC	-

**EGFP**	**Template**	**Forward**	**Reverse**	**RE for backbone**
Primer for fragment A	-	-	-	-
Primer for fragment B	-	-	-	-
**Men-CXXC-CHD-GFP**	**Template**	**Forward**	**Reverse**	**RE for backbone**
Primer for fragment A	Men-CXXC-CHD	GCATGGACGAGCTG TACAAG TAATGGAGCCCCAG GTTGATTCAATG	TCCTCGCCCTTGCTC AACATAGGTGTTTTG GTTAATTCTTGTAG	-
Primer for fragment B	EGFP	ATGTTGAGCAAGGG CGAGGAG	CTTGTACAGCTCGTC CATGCC	-
**N-MLL-GFP**	**Template**	**Forward**	**Reverse**	**RE for backbone**
Primer for fragment A	N-MLL	GTGCGAGGACCCCG GATTAAA	TCCTCGCCCTTGCTC AACATC TTTTCTTTTGGTTTTT GTTTTACAGGG	pciI
Primer for fragment B	N-MLL	GCATGGACGAGCTG TACAAG TAATGGAGCCCCAG GTTGATTCAATG	CTATCAGGGCGATG GCCCAC	draIII
Primer for fragment C	EGFP	ATGTTGAGCAAGGG CGAGGAG	CTTGTACAGCTCGTC CATGCC	-
**MLL_CHD-GFP**	**Template**	**Forward**	**Reverse**	**RE for backbone**
Primer for fragment A	MLL_CHD	GTGCGAGGACCCCG GATTAAA	TCCTCGCCCTTGCTC AACAT AGGTGTTTTGGTTAA TTCTTGTAG	pciI
Primer for fragment B	MLL_CHD	GCATGGACGAGCTG TACAAG TAATGGAGCCCCAG GTTGATTCAATG	GTCTATCAGGGCGA TGGCCCAC	draIII
Primer for fragment C	EGFP	ATGTTGAGCAAGGG CGAGGAG	CTTGTACAGCTCGTC CATGCC	-
**MLL-AF4_CXXCmut-GFP**	**Template**	**Forward**	**Reverse**	**RE for backbone**
Primer for fragment A	MLL-AF4_CXXCmut	GTGCGAGGACCCCG GATTAAA	CCTGGAGGGCTTTG TTTTAGAAGATTC	pciI
Primer for fragment B	MLL_CHD-GFP	CTAAAACAAAGCCCT CCAGG CCCTCCTCACAGTC CTCAAAGAA	CTATCAGGGCGATG GCCCAC	draIII

MLL-AF4_GFP	Template	Forward	Reverse	RE for backbone
Primer for fragment A	MLL-AF4	GTGCGAGGACCCCG GATTAAA	CCTGGAGGGCTTTG TTTTAGAAGATTC	pciI
Primer for fragment B	MLL_CHD-GFP	CTAAAACAAAGCCCT CCAGG CCCTCCTCACAGTC CTCAAAGAA	CTATCAGGGCGATG GCCCAC	draIII
